# The Immune Strategy and Stress Response of the Mediterranean Species of the *Bemisia tabaci* Complex to an Orally Delivered Bacterial Pathogen

**DOI:** 10.1371/journal.pone.0094477

**Published:** 2014-04-10

**Authors:** Chang-Rong Zhang, Shan Zhang, Jun Xia, Fang-Fang Li, Wen-Qiang Xia, Shu-Sheng Liu, Xiao-Wei Wang

**Affiliations:** Ministry of Agriculture Key Laboratory of Agricultural Entomology, Institute of Insect Sciences, Zhejiang University, Hangzhou, China; Kansas State University, United States of America

## Abstract

**Background:**

The whitefly, *Bemisia tabaci*, a notorious agricultural pest, has complex relationships with diverse microbes. The interactions of the whitefly with entomopathogens as well as its endosymbionts have received great attention, because of their potential importance in developing novel whitefly control technologies. To this end, a comprehensive understanding on the whitefly defense system is needed to further decipher those interactions.

**Methodology/Principal Findings:**

We conducted a comprehensive investigation of the whitefly's defense responses to infection, via oral ingestion, of the pathogen, *Pseudomonas aeruginosa*, using RNA-seq technology. Compared to uninfected whiteflies, 6 and 24 hours post-infected whiteflies showed 1,348 and 1,888 differentially expressed genes, respectively. Functional analysis of the differentially expressed genes revealed that the mitogen associated protein kinase (MAPK) pathway was activated after *P. aeruginosa* infection. Three knottin-like antimicrobial peptide genes and several components of the humoral and cellular immune responses were also activated, indicating that key immune elements recognized in other insect species are also important for the response of *B. tabaci* to pathogens. Our data also suggest that intestinal stem cell mediated epithelium renewal might be an important component of the whitefly's defense against oral bacterial infection. In addition, we show stress responses to be an essential component of the defense system.

**Conclusions/Significance:**

We identified for the first time the key immune-response elements utilized by *B. tabaci* against bacterial infection. This study provides a framework for future research into the complex interactions between whiteflies and microbes.

## Introduction

Insects interact and coexist with various types of microorganisms in many different ways and have evolved sophisticated strategies both to recognize and degrade entomopathogens, as well as to benefit from bacterial mutualists [1], [2]. Over 20% of all insect species are known to be associated with endosymbionts [3]. Amongst these, phloem-sap-feeding insects such as whiteflies, psyllids and aphids have been shown to possess specialized bacteriocytes that harbor primary and secondary endosymbionts [2]. These endosymbionts provide essential amino acids, protect the host from pathogen infection and also help it to adapt to different environments [3], [4], [5]. How these insects protect themselves from bacteria pathogens, while retaining beneficial endosymbionts, however, remains to be discovered [6]. Elucidating the host defense mechanisms of these insects will not only shed light on this question, but will also facilitate the development of novel insect-pest control strategies.

The whitefly, *Bemisia tabaci* (Gennadius) (Hemiptera: Aleyrodidae), is a cryptic species complex composed of at least 36 morphologically indistinguishable species [7], [8], [9], [10]. This species complex has members that rank as some of the most economically damaging insect pests [11]. *Bemisia tabaci* damages plants by direct sucking and by transmitting plant viruses [12]. In nature, whiteflies interact with various bacterial species, some of which are entomopathogenic and may serve as potential bio-control agents [13], [14]. Previous pyrosequencing analysis has revealed a diverse range of bacteria present in *B. tabaci*, though latest report showed the diversity of bacterial communities in *B. tabaci* is relatively limited [15], [16]. On the other hand, the primary (or obligatory) endosymbiont *Portiera* is considered to play a role in the synthesis of essential amino acids and carotenoids to the whitefly host [17], [18], while secondary endosymbionts may regulate the life parameters in various ways [19], [20]. These different interactions provide an excellent opportunity to study the immune system of an insect species that interacts simultaneously both with bacterial pathogens and endosymbionts. However, the molecular basis of the interactions between whitefly and those microbes remains largely unknown.

A previous study examined the transcriptome of whitefly exposed to the entomopathogenic fungus *Beauveria bassiana* and showed that only a limited number of canonical immune related genes were involved in the host's defense [21]. In addition, a genome-wide analysis and functional study showed that another hemipteran, the pea aphid, seems to lack many genes that are essential for the immune response in many other insects [22]. To investigate the immune system of Hemiptera further, we examined the gene expression profile of the whitefly *B. tabaci* under challenge from a well characterized Gram-negative bacterium *Pseudomonas aeruginosa* [23], [24], [25].

Most of the previous knowledge on insect immune response is based on host reactions after injection of bacteria into the insect's body cavity [1]. For most insects, however, the normal route of bacterial invasion is via oral ingestion [26], [27]. More recent work has shifted from cavity injection to systematic intestinal immune responses under oral infection, which mimics the natural mode of bacterial invasion [28]. Here, we examined the whitefly's essential host-defense strategies after oral bacterial challenge. Functional analysis of the differentially expressed genes indicate that MAPK cascade, antimicrobial peptides (AMP) and gut epithelium renewal play critical roles in the whitefly defense system, whereas stress responses are also induced to improve host tolerance.

## Materials and Methods

### Plants, whiteflies and bacteria cultures

The Mediterranean (MED) species of the *B. tabaci* complex was used in all the experiments [7]. A culture of MED was maintained on cotton plants (*Gossypium hirsutum* L. cv. Zhemian 1793) in climate chambers at 27±1°C, 14 h light/10 h darkness with 70±10% relative humidity (RH). The purity of the MED colony was checked every 5 generations by RAPDs and with sequencing of the mitochondrial cytochrome oxidase 1 gene [29],[30]. The *Pseudomonas aeruginosa* strain ATCC9027 was obtained from the Microorganisms Germplasm Bank of Guangzhou, China.

### Bioassay

Bioassays were carried out at 27±1°C and 70±10% RH. The infection solution was obtained from an overnight bacterial culture. The density of bacteria was adjusted to 1×10^8^ CFU/ml in 10% sucrose solution. The same sucrose solution without *P. aeruginosa* was used as control. Feeding and body cavity injection are two main approaches to carry out infection. In this study, the former method was chosen to mimic a natural mode of bacterial infection as well as to avoid physical damages to the whitefly. Transparent plastics tubes (L10, ø4 cm) were prepared as the feeding chambers for whiteflies. One end of the chamber was covered with a sandwich of 2 layers of carefully stretched Parafilm membrane separated by a layer of 1 ml bacterial solution. Approximately 100 newly emerged adult whiteflies were fed for 60 hours in each tube. The dead whiteflies were counted and cleaned out of the container every 6 hours. All treatments were replicated four times.

### Sample preparation for sequencing

Approximately 1,500 newly emerged adult whiteflies were collected for each treatment. At first, the control and 24 h treatment groups were fed, respectively, with sucrose and bacterial solution. After 18 hours, the 6 h treatment group was fed with bacteria. At 24 hours post-infection (hpi), approximately 1,000 whiteflies were collected from the control, 6 and 24 hpi treatments, respectively. This method ensured that the whiteflies were collected at the same developmental stage. In addition, because some whiteflies may not feed on the artificial diet at the beginning, the 6 hpi and 24 hpi whiteflies were fed with bacterial solution throughout the treatment to make sure that every whitefly individual can ingest enough bacteria. Samples were frozen immediately in liquid nitrogen and homogenated using the FastPrep system (MP Biomedicals). Total RNA was purified with SV total RNA isolation kit (Promega) according to the manufacturer's instructions. RNA quality was assessed by Nanodrop 2000 (Thermo Scientific) and 2100 Bioanalyzer (Agilent) as previously described [21], [31]. For each treatment, two biological replicates were conducted and processed independently. One replicate was used in the digital gene expression (DGE) library preparation and the other was used for real time quantitative PCR (qPCR) analysis.

### Digital gene expression (DGE) sequencing and tag annotation

The methodology for DGE sequencing was largely based on that described in previous studies [31], [32]. In brief, the mRNA from each sample was purified with magnetic oligo (dT) and subjected to cDNA synthesis. The cDNA was subsequently digested with *Nla*III, which recognizes the CATG sites. Then adapter 1 was ligated to the site of *Nla*III cleavage. The purified cDNA fragments were digested using *Mme*I that cuts 17 bp downstream of the CATG site, thus producing tags with adapter 1. Then, the adapter 2 was ligated at the site of *Mme*I cleavage. After 15 cycles of PCR linear amplification, 6% TBE polyacrylamide gel electrophoresis was used to purify the tags. After digestion, single strand molecules were added to the Illumina sequencing flowcell and fixed. The purified tags were sequenced by using Illumina HiSeq 2000 platform at the Beijing Genomics Institute (Shenzhen, China). DGE library data sets obtained from this work are available at the NCBI Gene Expression Omnibus under the accession number of GSE52837.

Clean tags were generated after removing 3′ adaptor sequences, low quality sequences, empty reads and tags with a copy number of 1. A reference database containing all possible CATG+17 nucleotide tag sequences were created for the transcriptome of the MED whitefly (unpublished data, available upon request). Sequencing tags were mapped to the whitefly transcriptome reference database with no more than one nucleotide mismatch. The number of unambiguous clean tags for each gene was calculated for gene expression analysis and TPM (number of transcripts per million tags) was used to normalize the data.

### Analysis of differentially expressed genes

The levels of gene expression were compared between: 1) the control library and the 6 hpi library; and 2) the control library and the 24 hpi library. False discovery rate (FDR)<0.05 and the absolute value of log_2_Ratio≥1 were used as the threshold to judge the significance of gene expression difference [33], [34]. Gene Ontology (GO) classification system provides a dynamic, controlled vocabulary for all eukaryotes [35] and was used to annotate the possible functions of differentially expressed genes (DEGs). Also, the Kyoto Encyclopedia of Genes and Genomes (KEGG) pathway analysis was used to depict the pattern of host response against bacterial challenge [36]. The number of DEGs in each GO term and KEGG pathway were also calculated. Using the MED transcriptome database as background, significantly enriched GO and KEGG pathway terms were determined using the hypergeometric test (*P*-value<0.05).

### Real time quantitative PCR (qPCR) analysis

To confirm the results of the DGE analysis, the expression of 20 selected genes was measured using qPCR. cDNA was synthesized using the SYBR PrimeScript reverse transcription-PCR kit II (Takara). qPCRs were performed in 96-well plates using the ABI Prism 7500 fast real-time PCR system (Applied Biosystems) with SYBR green detection. Each gene was analyzed in triplicate, after which the average threshold cycle (C_T_) was calculated per sample. The relative expression levels were calculated using the 2^−ΔΔCt^ =  2^−[ΔCt (treatment)− ΔCt (control)]^ method. The reference gene actin was used to normalize the expression level of other genes [37]. All of the designed primers were synthesized at Boshang BioCompany (Table S1).

## Results

### Whitefly survival curve

**Figure 1:**
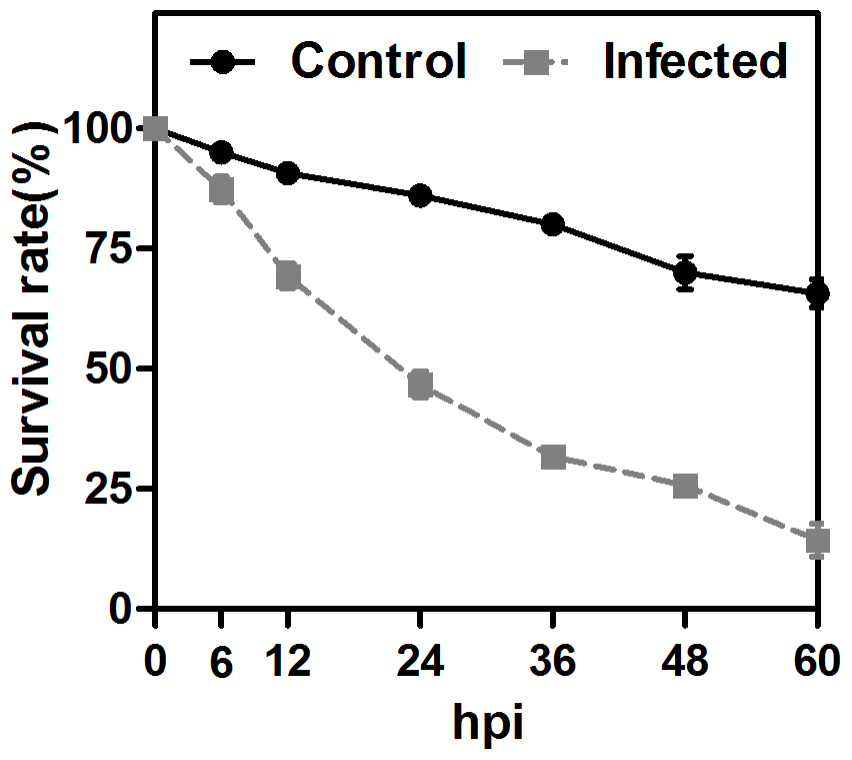
The survival curve of whitefly adults after infection of *P. aeruginosa* via oral ingestion. Error bars: ±SE of the mean.

This research focused on the response of whitefly to orally delivered bacteria; therefore it is important to find the important time points during bacterial infection. To achieve this, the survival rate of the whitefly was monitored for 60 h after *P. aeruginosa* ingestion. In this assay, a sharp decrease in the number of surviving whiteflies was observed during 12–36 hours post-infection (hpi) (Fig. 1). Therefore, 24 hpi was chosen as a sample collection time point. In addition, as sample collected earlier may reflect the whitefly's intestinal response to bacterial infection, the sample at 6 hpi was also collected for sequencing [26], [38].

### DGE library construction, sequencing and mapping

Three whitefly DGE libraries (Control, 6 hpi and 24 hpi) were sequenced and approximately 6 million raw tags were obtained for each library (Table S2). To determine the appropriate total tag sequencing number, a saturation analysis was carried out to check whether or not the number of detected genes kept increasing when the sequencing amount increased. Results for all three samples showed that when sequencing reached 5 million or higher, the increase in the number of detected genes was negligible (data not shown). After the removal of low quality reads, the numbers of distinct tags were 128641, 138701 and 126216 in the libraries of the control, 6 hpi and 24 hpi, respectively (Table S2). The ratio of clean tag to total tag was about 97% in each library. For annotation, the short tags of these DGE libraries were mapped to the MED whitefly transcriptome reference database. Tags which can map to more than one gene were filtered out. About 80% of clean tags were mapped unambiguously to the transcriptome database, showing the high quality of the sequencing and reference database. As a result, each library generated ∼17,000 tag-mapped genes, which also means that about 36% of genes in the whitefly transcriptome could be detected during DGE sequencing (Table S2).

### Differentially expressed genes (DEGs) and qPCR validation

**Figure 2:**
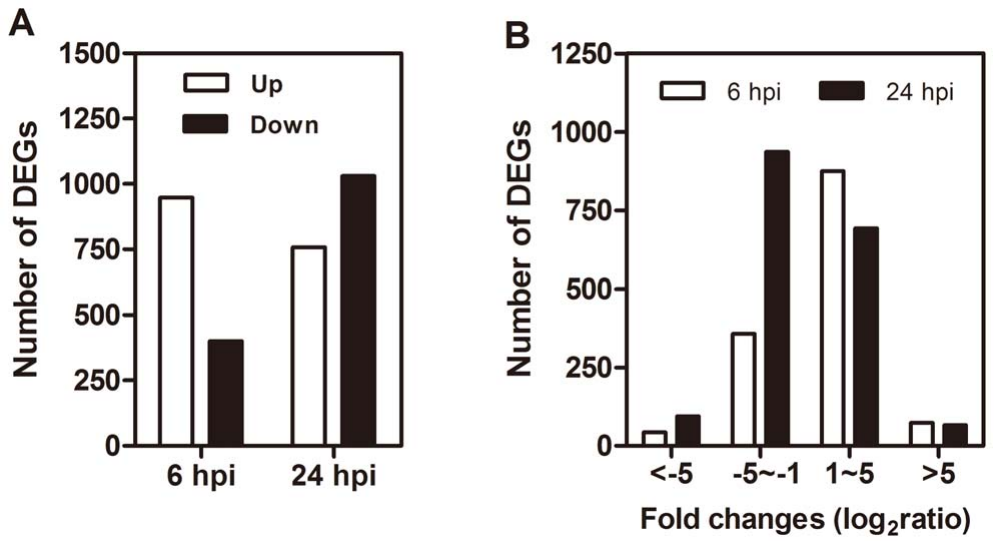
Analysis of differentially expressed genes. (A) An overview of differentially expressed genes (DEGs) between the whitefly libraries of 6 hpi and control, and of 24 hpi and control; the white and black bars indicate the up- and down- regulated genes, respectively. (B) The distribution of fold changes (log_2_ ratio) of the DEGs.

After annotation, we compared the control library with 6 hpi and 24 hpi bacterial challenged libraries to identify the DEGs that may play a central role in the host's defenses. Compared with the control, 948 genes were up-regulated and 400 genes were down-regulated at 6 hpi, while 758 genes were up-regulated and 1030 genes were down-regulated at 24 hpi (Fig. 2A, Table S3). Interestingly, at 6 hpi, the majority of DEGs were up-regulated. At this time point, the mortality of infected whiteflies started to increase, which indicated that the whiteflies had come into close interaction with the bacteria and had responded to pathogen infection. Therefore, 6 hpi reflects the early phase of whitefly defense against *P. aeruginosa*. At the 24 hpi, MED death increased rapidly. The bacteria had already clearly imposed substantial stress on the host. As a result, the sample at 6 hpi and 24 hpi represent different stages of MED's defense response.

The detected fold changes (log_2_ ratio) of gene expression ranged from −9.57 to 9.57, and the majority of genes were up- or down-regulated between 1.0- and 5.0-fold, respectively (Figure 2B). In previous studies, the DGE method has been proven to have high reliability [31], [39], [40]. Due to the relatively high expense of Illumina sequencing, only one sequencing run was performed for each sample. To validate the DGE data, 20 selected genes were quantified for their transcription levels with qPCR in the 6 hpi and 24 hpi treatments. In 34 out of 40 tests, qRT-PCR results were consistent with the DGE data, providing further evidence of the reliability of our sequencing results (Table S1).

### Ingestion of bacteria caused transcriptome reprogramming

Gene Ontology (GO) and Kyoto Encyclopedia of Genes and Genomes (KEGG) pathway analysis are widely used to examine the biological processes in a large group of genes [35], [36]. First, different GO and KEGG Orthology (KO) terms were assigned to DEGs. In the 6 hpi and 24 hpi data, 738 and 965 DEGs had GO annotations while 187 and 283 genes were annotated with KEGG terms. Enriched GO and KO terms in DEGs were then identified using hypergeometric analysis with the MED transcriptome database as background (Tables S4 and S5). In GO enrichment analysis, 124 and 136 GO terms under the category of biological process were over-presented in the 6 hpi and 24 hpi libraries, respectively (Table S4). At both time-points, cell cycle, cell proliferation, stress responses, genetic information processing and metabolic processes featured among the enriched GO terms. KEGG pathway enrichment analysis showed similar pattern and 21 and 17 pathways were enriched at 6 hpi and 24 hpi, respectively (Table S5). More importantly, the phagocytosis and melanogenesis related pathways were enriched in DEGs suggesting the critical role of the whitefly's cellular immune responses during bacterial infection. The involvement of multiple GO and KEGG pathways indicated that *P. aeruginosa* infection activated a number of cellular and molecular responses, which is discussed below.

### Microbial recognition and signal transduction pathways

Pathogen recognition is the initial event of pathway activation and systematic responses. Host defense responses are initiated when microbial molecules such as lipopolysaccharides and peptidoglycans are detected by pattern-recognition receptors (PRRs)[1]. Among those receptors, c-type lectins are sugar binding proteins specifically binding to polysaccharide chains on the pathogen's surface [41]. In infected whiteflies, two c-type lectins were up-regulated in both 6 hpi and 24 hpi treatment groups (Table 1). C-type lectins have been shown to enhance hemocyte encapsulation ability in cellular immunity and activate prophenoloxidase in humoral immunity [42]. Thus, MED c-type lectins are probably able to regulate cellular and humoral immunity upon bacterial infection.

**Table 1:** Genes involved in immune related signaling^a^.

Gene	Homologous function^b^	Accession	FC6^c^	FC24
**c-type lectin**				
comp29769_c0	C-type lectin like	XP_001989543.1	1.40	0.78
comp35820_c0	C-type lectin	EFA04178.1	0.83	1.28
**MAPK**				
comp38361_c0	TAK1-binding protein 1	XP_001640361.1	1.29	1.24
comp52776_c0	Afadin	XP_003736747.1	1.57	1.13
comp28150_c0	Insulin receptor	EGI60406.1	6.64	6.32
comp34866_c0	Ribosomal protein S6 kinase alpha-1	XP_001504130.1	2.39	2.09
comp31255_c0	MAPKKK13/LZK	XP_002068236.1	2.04	1.09
comp38783_c0	TRB2 protein	AAP04410.1	1.50	0.78
comp40004_c0	Raf serine/threonine-protein kinase	XP_001355538.2	1.06	0.94
comp22807_c0	Neurofibromin	EFZ16398.1	4.91	7.02
comp35826_c0	MAPKKK7/TAK1	ABY81296.1	0.89	1.48
comp34358_c0	HSP70	ADK94698.1	−1.04	−1.48
comp31282_c0	cheerio, isoform I	NP_001189238.1	−1.23	−1.51
comp21240_c0	HSP68	NP_001243928.1	−2.45	−4.70
comp31513_c0	Camp-dependent protein kinase 3	JAA55823.1	−1.04	−2.21
**JAK-STAT**				
comp39622_c0	CREB binding protein/P300	AAB53050.1	1.30	1.50
comp34743_c1	Phosphoinositide 3-kinase	XP_002001080.1	1.74	1.35

a The genes with fold change >2 fold (|log_2_ratio|>1) and FDR <0.05 are considered to be significant.

b Homologous function: the function of the homologous gene.

c FC: fold change (log_2_Ratio) of gene expression, where ratio  =  TPM (6 or 24 hpi)/TPM (control). Underlined fold-change values represent significant changes to differentially expressed genes at given time point.

Activation of signaling pathways follows pathogen recognition. The MAPK pathway, which comprises the ERK, JNK and p38 mediated kinase cascade, is a conserved insect host-defense repertoire [43]. Dysfunctions of JNK and p38 resulted in hypersensitivity toward infection and stress [44], [45]. Here, several essential MAPK components were activated, including MAP3K7/TAK1, its binding protein TAB1 and MAP3K13/LZK (Table 1). TAK1 is required for the activation of NF-κB and JNK pathways in *Drosophila*, while LZK is able to phosphorylate and activate JNK [46], [47], [48]. More interestingly, a putative peroxiredoxin was suppressed at 24 hpi and its homolog in *Drosophila* was identified as a negative regulator in the Tak1-JNK arm of the immune signaling system (Table S3) [49]. In addition, other genes associated with MAPK were activated, such as an insulin/growth factor receptor, a ribosomal protein S6 kinase and tribbles homolog 2. The findings provide additional evidence of the importance of the JNK pathways following *P. aeruginosa* infection. Moreover, the CREB binding protein/P300 and phosphoinositide 3-kinase, two players in JAK/STAT pathways, were also up-regulated significantly (Table 1).

### Activation of AMPs and other effectors in immunity

**Figure 3:**
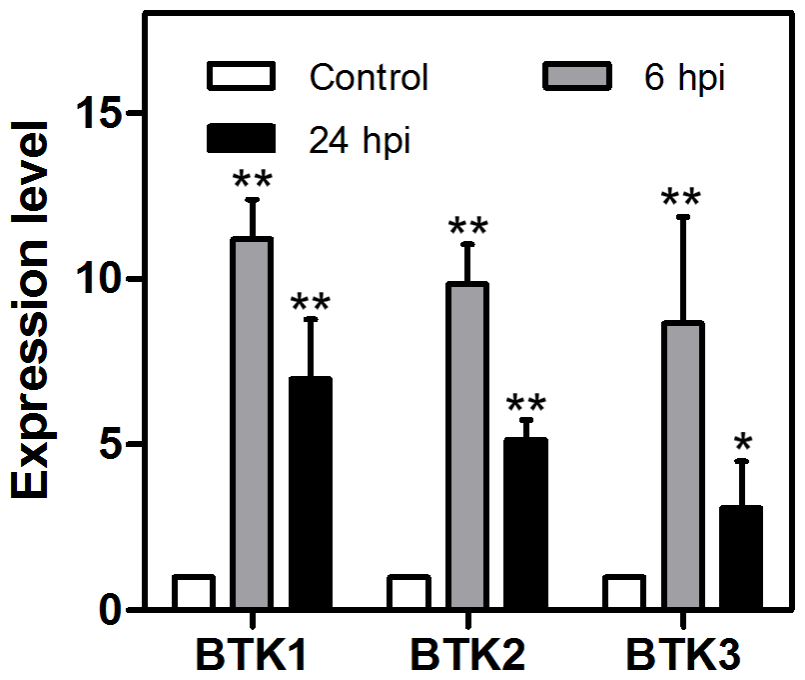
The relative expression level of BTK1, BTK2 and BTK3. The expression level of these genes in uninfected whiteflies was set to 1 (white). Grey and black represent 6 hpi and 24 hpi respectively. Statistical significance compared with Control of P≤0.05 (*) and P≤0.001 (**), Student's *t* test. Error bars: ±SE of the mean.

Fast and massive production of AMPs has evolved in insects to be a central strategy of their immune system. Knottins are small proteins that have antimicrobial peptide activities and they are widely present in both plants and insects [50]. The DGE analysis revealed that three antimicrobial knottin genes (Btk 1, 2, and 3) were induced at 6 hpi and this was confirmed subsequently by the qPCR data (Table 2, Fig 3). In *Drosophila*, TAK1 and its binding protein are able to activate the NF-κB pathway and, ultimately, the production of AMPs [51], [52]. Whether this signaling pathway is conserved in *B. tabaci*, however, remains to be discovered. Other than activation of AMPs, coagulation and melanization are also important for microbe sequestration and degradation. In insects, serine proteases and their inhibitors (serpin) are responsible for the activation and regulation of coagulation and melanization [53], [54]. Several serine proteases are modulated in *P. aeruginosa* infected whiteflies. Interestingly though, 1 and 2 serine proteases are up- and down-regulated at 6 hpi, whereas 2 and 6 serine proteases are clearly up- and down-regulated at 24 hpi, respectively (Table 2).

**Table 2:** Genes involved in humoral immunity.

Gene	Homologous function	Accession	FC6	FC24
**AMP**				
comp28129_c0	Btk-1	ACT78451.1	1.62	0.47
comp33051_c0	Btk-2	ACT78451.1	1.61	0.27
comp31823_c0	Btk-3	ABC40571.1	1.90	0.67
**Serine proteases**				
comp39743_c0	Putative serine protease	EFR26537.1	1.34	1.35
comp20520_c0	Serine proteinase stubble	XP_001989504.1	−0.42	1.62
comp38353_c0	Putative serine protease	XP_002035636.1	−1.85	−1.83
comp42030_c0	Putative serine protease	EKC26449.1	−3.09	−7.41
comp32453_c0	Putative serine protease	XP_001653636.1	−0.53	−1.03
comp33254_c1	Putative serine protease	XP_002593630.1	0.18	−1.12
comp31974_c0	Putative serine protease	XP_783667.2	−0.93	−2.40
comp36335_c1	Putative serine protease	XP_002593630.1	−0.52	−2.94

### Regulations of cell proliferation and epithelium renewal upon infection

The intestinal epithelium is the first protective barrier of the host from orally delivered microorganism infection. In *Drosophila*, stem cell division was activated upon infection to compensate for the cell damage due to the presence of bacteria and also to maintain gut homeostasis [38], [55], [56], [57], [58]. The enrichment of GO terms such as cell cycle (GO:0007049), positive regulation of cell proliferation (GO:0008284) and epithelium development (GO:0060429) indicated the involvement of cell proliferation and epithelium renewal in host defenses (Table S4). About 80% of genes were induced at both 6 hpi and 24 hpi and extensive genetic studies on the *Drosophila* gut have shown that this process is governed by JNK, JAK-STAT, Wingless and Epidermal growth factor receptor (Egfr) pathways [55], [56], [59], [60], and all of these pathways were also regulated in our data. In addition, a Wingless like protein and several related genes in the Egfr pathway were induced (Table 3; Cordero et al, 2012; Jiang et al, 2011; Xu et al, 2011). Several other well-characterized genes in cell proliferation were also activated, including SMAD1, SMAD4 and an insulin receptor (Table 3).

**Table 3:** Genes involved in cell proliferation and related pathway.

Gene	Homologous function	Accession	FC6	FC24
**Cell proliferation**				
comp34792_c3	SMAD4	XP_003227329.1	1.52	1.14
comp28150_c0	Insulin receptor	EGI60406.1	6.64	6.32
comp35213_c0	Paired box protein Pax-6	ABS17534.1	3.25	1.58
comp34974_c0	Lysine-specific histone demethylase	XP_003814021.1	1.01	1.00
comp40004_c0	Raf serine/threonine-protein kinase	XP_001355538.2	1.06	0.94
comp38386_c0	SMAD1	NP_001259992.1	1.13	2.06
comp31937_c0	NADH dehydrogenase	XP_001977774.1	−1.06	−0.87
**Wnt pathway**				
comp35633_c0	wnt11	NP_001192557.1	1.44	0.20
**Egfr pathway**				
comp38516_c0	kekkon5, isoform A	NP_573382.1	3.32	3.17
comp37205_c0	fusilli-like	XP_002736133.1	1.38	1.05
comp35265_c0	Protein mago nash	EHJ65953.1	0.27	1.06
comp20819_c0	AP-2 complex subunit sigma	EGW03559.1	−0.34	−1.20

### Stress response genes of whitefly

The high motility caused by *P. aeruginosa* clearly showed that a hyper-biotic stress had been imposed on the whitefly by 24 hpi. DEGs were consequently highly enriched in the GO term: regulation of response to stress (GO: 0080134) at both 6 hpi and 24 hpi (Table S4). This result is consistent with the activation of the MAPK, because their roles in the stress response are widely accepted [43].

Chaperones work in protein folding and quality control, which maintains cellular homeostasis and buffers environmental stress. Our analysis showed that several chaperones and cofactors were regulated. Five out of seven genes were induced at 6 hpi whereas five out of six genes were repressed at 24 hpi (Table 4). Induced genes include several cofactors of HSP90 and HSP40 proteins. In addition, a large group of genes involved in detoxification, such as Cytochrome P450, Glutathione S-transferase and Glutathione peroxidase were also regulated (Table 5). These genes are likely involved in the detoxification of reactive oxygen species, as well as other toxins produced by bacteria [61], [62]. Interestingly, 15 out 16 Cytochrome P450 genes were suppressed at 24 hpi. Cytochrome P450 genes were also suppressed following pathogen infection [56], [63]. It suggests there is a general trend of P450 suppression after pathogen infection in other insect species.

**Table 4:** Genes involved in protein folding and DNA repair.

Gene	Homologous function	Accession	FC6	FC24
**Chaperones**				
comp37167_c0	YLP motif-containing protein 1	EHB15431.1	1.05	1.16
comp40894_c0	HSP90 co-chaperone CPR7	XP_002105226.1	2.25	0.07
comp39790_c0	HSP90 co-chaperone CPR7	XP_002105226.1	1.29	0.51
comp38394_c4	Hsp90 co-chaperone/Cdc37	XP_002046556.1	1.05	0.76
comp37509_c0	DnaJ subfamily C member 14	XP_001978829.1	1.04	0.75
comp34358_c0	HSP70	ADK94698.1	−1.04	−1.48
comp21240_c0	HSP68	NP_001243928.1	−2.45	−4.70
comp40216_c0	beta-tubulin folding cofactor C	XP_002735209.1	−0.51	−1.19
comp13402_c0	DnaJ subfamily C member 19	XP_003488785.1	−0.75	−1.39
comp20054_c0	molecular chaperone DnaJ	XP_003248597.1	−0.45	−1.46
**DNA repair**				
comp30427_c0	G/T mismatch-specific thymine DNA glycosylase	XP_002011422.1	1.25	1.54
comp37437_c0	DNA repair protein XRCC2-like	XP_003134584.1	1.19	1.13
comp35539_c0	DNA-repair protein XRCC3	NP_001079887.1	1.38	0.66
comp32492_c0	Predicted methyltransferase	XP_001986788.1	1.04	0.04
comp39604_c0	DNA repair protein RAD18	EFZ21621.1	1.91	2.39
comp29666_c0	Nucleotide excision repair complex XPC-HR23B, subunit XPC/DPB11	CAA82262.1	0.93	1.45
comp31941_c0	DNA repair protein RAD50 isoform 1	XP_004042521.1	0.89	1.38
comp40044_c0	DNA repair protein REV1	NP_612047.1	0.63	1.12
comp38825_c1	DNA mismatch repair protein Msh2	XP_003473123.1	0.53	1.10
comp33285_c0	DNA damage-responsive repressor GIS1/RPH1	—	−2.03	−1.19

**Table 5:** Differentially expressed detoxification enzymes.

Gene	Homologous function	Accession	FC6	FC24
**Glutathione S-transferase**				
comp35665_c0	glutathione S-transferase	EFA01955.1	1.16	0.63
**Glutathione peroxidase**				
comp36914_c0	glutathione peroxidase	EFX89084.1	−0.29	−1.25
**Cytochrome P450**				
comp36389_c0	cytochrome P450	XP_001865029.1	1.77	0.85
comp33887_c0	Predicted similar to cytochrome P450	XP_966563.2	1.24	0.09
comp30063_c1	cytochrome P450	EFR21005.1	1.02	−0.96
comp402849_c0	cytochrome P450	XP_001987651.1	6.32	6.64
comp35292_c0	CYP6M1a	AFM08393.1	−1.09	−2.01
comp35528_c0	cytochrome P450	AEK21822.1	−1.12	−1.77
comp35166_c0	cytochrome P450 6BQ5	EFA02819.1	−1.17	−1.25
comp35935_c1	cytochrome P450 CYP6BK17	XP_969633.1	−1.47	−2.89
comp24705_c0	cytochrome P450	AEK21822.1	−1.49	−2.32
comp37361_c0	cytochrome P450	AEK21804.1	−1.69	−1.93
comp320941_c0	cytochrome P450 345C1	EFA12854.1	−1.26	−6.91
comp38461_c0	cytochrome P450	XP_966391.1	−0.21	−1.04
comp37745_c0	cytochrome P450	EFA02819.1	−0.05	−1.06
comp36384_c0	cytochrome P450	AFP49818.1	−0.74	−1.43
comp29930_c0	cytochrome P450 6a2-like isoform 2	XP_003248187.1	−0.15	−1.77
comp27263_c0	cytochrome P450 345C1	EHJ67475.1	−0.45	−1.78
comp37119_c0	cytochrome P450 protein	NP_001156683.2	−0.95	−1.95
comp37634_c1	cytochrome P450 6BQ13	EEZ99338.1	−0.25	−2.03
comp40428_c0	cytochrome P450	XP_001653674.1	−0.73	−2.73

Another notable event in infected whitefly was the activation of DNA repair proteins. Five and seven DNA repair genes were induced in 6 hpi and 24 hpi whitefly, respectively (Table 4). The only repressed gene was a negative regulator in DNA damage repair (Table 4) [64]. DNA damage caused by pathogenesis might have triggered the DNA repair response, because several genes in mismatch repair and double-strand break response have been reported to be highly activated to improve host defense [65]. An alternative explanation, however, is that these genes might also participate in cell-cycle checkpoint, as our data also show that cell proliferation is activated [66].

### Modulation of basal metabolism

**Figure 4:**
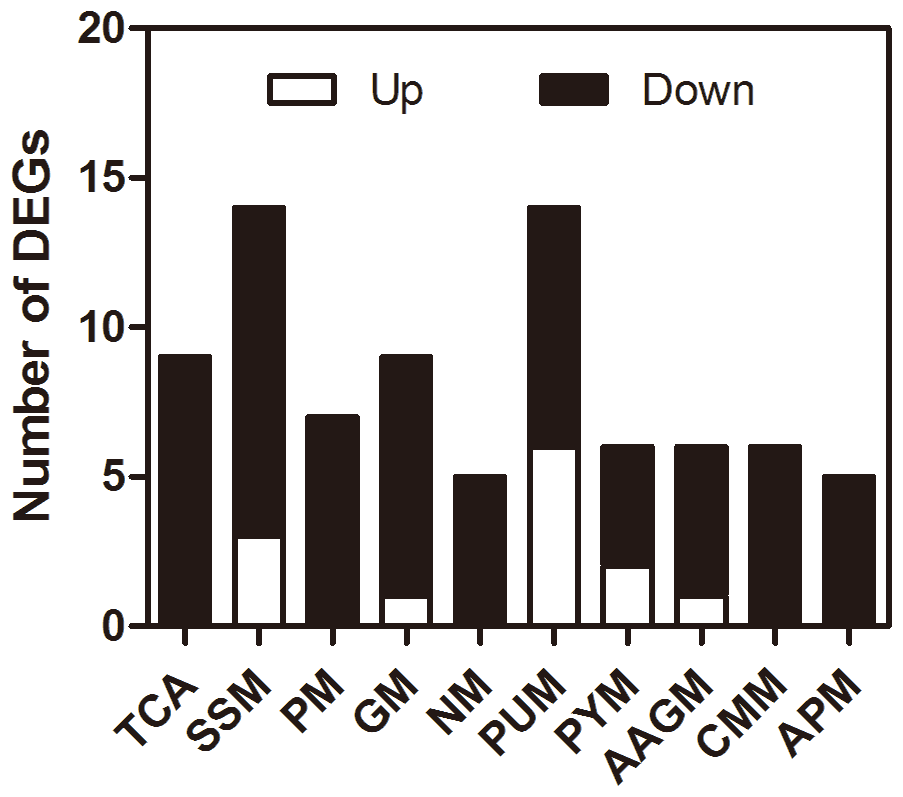
Modulation of basal metabolism-related genes in 24 hpi whiteflies. The numbers of up- and down-regulated genes are shown in white and black, respectively. The listed metabolism pathways are: TCA cycle (TCA, ko00020), Starch and sucrose metabolism (SSM, ko00500), Pyruvate metabolism (PM, ko00620), Galactose metabolism (GM, ko00052), Nitrogen metabolism (NM, ko00910), Purine metabolism (PUM, ko00230), Pyrimidine metabolism (PYM, ko00240), Alanine, aspartate and glutamate metabolism (AAGM, ko00250), Cysteine and methionine metabolism (CMM, ko00270), Arginine and proline metabolism (APM, ko00330).

Orally-delivered infection of *P. aeruginosa* also modulated a large group of basal metabolism processes, especially in the late infection stage. Given that intestinal function is disturbed in pathogenesis, these regulations may act as a self-protective strategy to maintain and reallocate energy supply. Our data shows that the majority of metabolism-related genes were down-regulated at 24 hpi (Fig 4). Suspension of feeding has been discovered in other insects that encountered bacterial infection and thus the inhibition of metabolism might be a direct result of this [67]. Whitefly may have evolved this adaptive strategy to prevent further pathogen ingestion. In addition, this down-regulation might be due to the disruption of energy supply under bacterial infection.

## Discussion

Despite its economic importance as a global pest, little is known about the immune response of the *B. tabaci* complex to bacterial infection. Recent developments in next-generation RNA-seq technology, however, allow a systematic study of the whitefly's host defense strategies upon intestinal bacterial infection. In whiteflies collected at both time points following bacterial infection, more than one thousand genes were modulated. Functional analysis uncovered the complexity of the host's responses to pathogenic bacteria. Bacterial infection not only induced genes in immune signaling and several types of effectors, but also altered the expression pattern of several sets of genes in xenobiotics detoxification, protein folding, DNA repair and basal metabolism. Altogether, our analysis provides the first rough outline of the whitefly's defense mechanisms employed against pathogenic bacteria and raises further questions that deserve more investigation.

The major goal of our research was to decipher the immune strategies of *B. tabaci*. Humoral and cellular immunity are the two arms of the insect's defense system, and in the former response antimicrobial peptides (AMPs), enzymatic cascades and other soluble effectors are employed to degrade foreign invaders [1]. In our study, both DGE data and qPCR analysis showed the activation of AMP production, highlighting again its core status in the antimicrobial response. As with the cellular immune system, pathway analysis showed clearly the involvement of phagocytosis and melanogenesis, more research is needed to construct a comprehensive image. In addition, our data implicates the function of intestinal stem cell proliferation in gut homeostasis maintenance, which may be conserved in whitefly.

The signaling pathways governing immune processes were also revealed by our work. We observed significant activation of several arms of MAPK cascades. Raf and ribosomal protein S6 kinase are essential kinases in the ERK pathway, while TAK1, TAB1 and TRB2 can control both p38 and JNK pathways [43], [68]. Studies in *Drosophila* showed TAK1 and TAB are located at the cross point of the JNK and Imd pathway in fruit fly [51], [52]. Upon activation, TAK1 and TAB can regulate NF-κB and then activate AMP production. Ras/MAPK signaling can also mediate intestinal homeostasis and regeneration in *Drosophila*. Exploring downstream events controlled by MAPKs, therefore, may provide key answers to how those processes are regulated in whiteflies. Besides the MAPK pathway, only a few genes in other canonical pathways were identified in infected whiteflies. For instance, we failed to identify the canonical factors in the Imd pathway from whiteflies. Interestingly, the pea aphid, another hemipteran insect, also appears to lack the Imd pathway [22]. Nevertheless, the absence of these genes may be due to the limitation of our reference database, which only accounts for a part of the *B. tabaci* genome. Likewise, although several antimicrobial knottins were induced, their induction level was relatively modest compared to that of other insects. Surprisingly, several defensins were not up-regulated upon pathogen infection (data not shown). More analysis is needed to characterize the function of whitefly AMPs.

Our analyses also enable a close examination of the stress response strategy of *B. tabaci*. Though stress response genes are not involved directly in immunity, their importance in the host's defense systems is recognized. Activation of these genes can help the host maintain cellular homeostasis and increase its capacity to endure the infection [69]. The involvement of chaperones, detoxification enzymes and DNA damage repair is likely to help the whitefly build up a high tolerance towards infection. In fact, these genes also participate in environmental adaptation and the development of resistance to insecticides [70], [71]. An understanding of the regulation of these gene sets in particular may help in the development of novel insecticides.

In summary, we report for the first time the results of an NGS investigation into the molecular interactions induced by the oral delivery of a bacterial pathogen, *P. aeruginosa*, to the whitefly *B. tabaci*. Functional analyses of DEGs indicated that at 6 hpi both humoral and cellular responses are involved in the whitefly's defense responses. Furthermore, MAPK cascade, AMP and gut epithelium renewal probably play critical roles in the defense system, whereas stress response genes are also induced to build stronger host tolerance. Of particular interest is that only a few genes in other canonical pathways were identified in infected whiteflies. Further research built upon these findings may present an opportunity for the development of a novel whitefly control technologies.

## Supporting Information

Table S1 **qRT-PCR primers and results.**
(XLSX)

Table S2 **Overview of the DGE sequencing results.**
(DOC)

Table S3 **List of the differentially expressed genes.**
(XLS)

Table S4 **Results of the Gene Ontology enrichment analysis.**
(XLS)

Table S5 **Results of the KEGG pathway enrichment analysis.**
(XLS)
