# Supplementary material for: The Immune Strategy and Stress Response of the Mediterranean Species of the Bemisia tabaci Complex to an Orally Delivered Bacterial Pathogen
Source: PLoS One. 2014 Apr 10;9(4):e94477. doi: 10.1371/journal.pone.0094477 (PMC3983193; doi:10.1371/journal.pone.0094477)
Supplement: Table S2 — Overview of the DGE sequencing results. (DOC) [file pone.0094477.s002.doc]

| Category | Parameter | Whitefly library | | | | |
| --- | --- | --- | --- | --- | --- | --- |
|  |  | Control | 6 hpi | | 24 hpi | |
| Clean Tag | Total number | 5985368 | | 6063303 | | 6036983 |
|  | Distinct Tag number | 128641 | | 138701 | | 126216 |
| Unambiguous Tag Mapping to Gene | Distinct Tag number | 105392 | | 113458 | | 100668 |
|  | Distinct Tag % of clean tag | 81.93% | | 81.80% | | 79.76% |
| Unambiguous Tag-mapped Genes | number | 17153 | | 17785 | | 17020 |
|  | % of ref genes | 36.65% | | 38.00% | | 36.36% |

**Table S2. Overview of the DGE sequencing results**
